# Health promoting lifestyle behaviors and associated predictors among clinical nurses in China: a cross-sectional study

**DOI:** 10.1186/s12912-021-00752-7

**Published:** 2021-11-17

**Authors:** Wen Zeng, Shaomei Shang, Qian Fang, Shan He, Juan Li, Yuanrong Yao

**Affiliations:** 1 Nursing School of Peking University Health Science Center, No.38, Xueyuan Road, Haidian District, 100191 Beijing , Beijing City China; 2 Guizhou Provincial People’s Hospital, No.83 Zhongshan East Road, Nanming District, 550002 Guiyang, China

**Keywords:** Health Promotion, Nurses, Health Behavior, Self Care

## Abstract

**Background:**

Nurses play a core role and encompass the main workforce in health care systems. Their role model of health promoting lifestyle behaviors (HPLB) would directly or indirectly affect their clients’ beliefs or attitudes of health promotion. There is limited evidence on HPLB in clinical registered nurses. The current study aimed to explore the HPLB and associated influencing factors among clinical registered nurses in China.

**Methods:**

A multi-center cross-sectional anonymous online survey was conducted in 2020. Participants were asked to complete social demographic information as well as the revised Chinese edition of Health Promoting Lifestyle Profile (HPLP). Independent-Sample T-Test, One-Way ANOVA, and categorical regression (optimal scaling regression) were the main methods to analyze the relationship between demographic data and the score of HPLB.

**Results:**

19,422 nurses were included in the study. The mean score of self-actualization, health responsibility/physical activity, nutrition, job safety, interpersonal support, and overall Health Promoting Lifestyle Profile were, 27.61(5.42) out of a score of 36, 22.71(7.77) out of a score of 44, 10.43(2.97) out of a score of 16, 22.05(3.97) out of a score of 28, 20.19(4.67) out of a score of 28, and 102.99 (19.93) out of a score of 144, respectively. There was a significant relationship among Hospital levels, working years, nightshift status, and monthly income per person, and mean score of all subscales and the overall HPLP (*P* < 0.05).

**Conclusions:**

Nurses who participated in the study presented a moderate level of health promoting lifestyle behaviors. Hospital levels, working years, nightshift status, and monthly income per person were predictors for all subscales and overall HPLP.

## Background

Health is a global theme. More empirical evidence suggests that it is much more important to take individuals’ capacity to promote their health into consideration rather than to treat disease [1]. The rapidly growing evidence indicates that individuals could take measures to promote their health and well-being [2]. Health Promotion (HP) is one of the core determinants and strategies for individuals to facilitate and maintain their health [3], and it has become of global importance [4]. In terms of HP, it has been defined in various ways and the World Health Organization (WHO) defined it as “the process of enabling people to increase control over, and to improve, their health” [5]. In recent years, there is a great emphasis on Health Promoting Lifestyle Behaviors (HPLB). HPLB, which is one of the key concepts in the nursing field [6], is defined as individuals’ actions and beliefs on multiple levels of HP including self-actualization, physical activities/health responsibility, food choices, interpersonal support [7] to enhance or preserve the level of well-being or reduce the incidence of illness [8].

Nurses play a core role and encompass the main workforce in healthcare systems. According to the statistics of WHO, there are 43.5 million health workers worldwide, while nurses are more than 20 million [9]. Notably, more than 4 million registered nurses are in China [10]. In other words, Chinese nurses account for nearly one-fifth of global nurses. Nurses deliver diverse healthcare services in different settings as health care providers, protectors, communicators, coordinators, decision-makers, and teachers [11]. In fact, nurses are usually the first responders to different health-related conditions to promote health and rehabilitation, and prevent disease [11]. More important, Nurses usually act as a role model, a leader as well as an HP practitioner for the public in empowering and motivating individuals and communities to engage in HP decision-making, HP action and activities to promote their health condition [3, 12, 13]. Empirical evidence shows that nurses’ own health practices could largely produce an effect on the effectiveness of the health intervention provided for their clients [8].

The importance of nurses’ role in HP has been largely reported, it is believed that nurses’ role model of HP behaviors would directly or indirectly affect their clients’ beliefs or attitudes of HP [12]. Therefore, it is important to know the level of HPLB in nurses. To our best knowledge, most studies on HPLB focus on nursing students [7, 8, 13–16] or patients [17]. There is limited evidence on HPLB in clinical registered nurses. Besides, most measurements used to examine individual HPLB do not contain the aspect of job security. Therefore, a modified Health Promoting Lifestyle Profile (HPLP) would be used to examine the HPLB of clinical nurses in China. Additionally, the current study aimed to explore the associated factors that influence the level of HPLB.

## Methods

### Design, setting and participants

A multi-center cross-sectional survey was carried out from January 8^th^ 2020 to January 30^th^ 2020 in 42 hospitals from 26 cities, 16 provinces in China. These hospitals, which were representative of different levels of healthcare services, were including primary, secondary and tertiary institutions. Registered nurses working as staff nurses in the selected hospital were included. Nursing students and retired nurses were excluded.

The investigation was completed online. Firstly, researchers imported the informed consent agreement and the questionnaire of the revised Chinese edition of HPLP [18] on an online survey plat, Chinese Questionnaire Star (https://www.wjx.cn/), then freely created a QR code. Researchers released the QR code through a social media application WeChat. Participants could scan the QR to read and submit the informed consent agreement. After that, they could choose to fulfill the questionnaire. Each participant was only allowed to submit once in order to avoid double submission.

### Variables and assessment

Independent variables in the study were some sociodemographic characteristics including gender, age, basic education background, highest education background, hospital level, working years, nightshift status, working department, marital status, number of children, living with parents, monthly income per family member.

In order to evaluate the level of nurses’ HPLB, a modified Chinese edition of HPLP [18] was used in the study. The instrument, which was originally developed by Walker, Sechrist and Pender [19] and translated into the Chinese edition by Chen et al. [20]. Based on the Chinese edition of HPLP, Sun, Huang and Ling [18] developed a modified Chinese edition of HPLP which consists of 5 subscales (38 items), namely, self-actualization (9 items), health responsibility/physical activities (11 items), nutrition (4 items), job security (7 items) and interpersonal support (7 items). The instrument is a 4 Likert scale and the score of each item ranges from 1 to 4: 1 = never, 2 = sometimes, 3 = often, 4 = routinely. The overall score of the HPLP ranges from 38 to 144. A higher score represents better HPLB. The Cronbach’s alpha was 0.9278, and 3 weeks of test-retest reliability Cronbach’s alpha was 0.818.

### Data collection

Two researchers who didn’t know the study design collected the data. Before the data collection, those two researchers received training courses about how to collect the data, check the data, input and code the data into IBM SPSS 25.0. After the training courses, they took part in an exam related to data collection. Only if they passed through the exam could they be involved in collecting data.

### Data analysis

All data were imported into IBM SPSS 25.0. Mean (standard deviation, SD) was used to describe the continuous data. Categorical variables were presented as frequency (N) and percentages. To investigate the potential factors associated with HPLP, a univariate analysis was conducted first to examine the difference of mean scores among groups. At this stage, Independent-Sample T-Test and One-Way ANOVA were performed. Secondly, categorical regression (optimal scaling regression) was the main method to analyze factors that had been confirmed to be statistically significant in the univariate analysis in order to test the correlation between the demographic data and HPLP. In the current study, the *P* value was two-tailed and we inferred statistical significance if α was less than 0.05.

## Results

### Demographic characteristics of the participants

19,522 nurses completed the questionnaires. However, 100 questionnaires were removed from the analysis because of the following reasons: (1) Responders’ age was logically incorrect; (2) Participants’ working years were more than their age. Therefore, the effective response rate was 99.49 %.

As shown in Table 1, female nurses dominated the main responders (95.66 %). Participants’ age ranged from 18 to 62 years old. Most (55.11 %) of the included nurses were with an associate degree, while only 4531 nurses (23.33 %) originally graduated from college or university and got a bachelor’s degree. However, 54.91 % of the included nurses finally got a bachelor degree after receiving continuous education. 10,487 nurses were working at a secondary level of hospital and 8619 nurses were at a tertiary hospital. In the current study, the working years of nurses ranged from 1 to 41 years. Nurses who worked nightshift were more than 55 %. Besides, nurses on the schedule of nightshift working were younger, less working age, mainly never married, less educated than those without nightshift. And more than 70 % of the respondents worked at the Inpatient General Department. 68.02 % of the nurses were married and 28.71 % were never married. Only 36.75 % of the nurses reported without any child. Nearly 70 % of the respondents reported a monthly income per family member of more than 2000 RMB (¥2000).

**Table 1:** Demographic data of the participants

	Frequency	Percentage
**Gender**		
Male	842	4.34 %
Female	18,580	95.66 %
**Age**		
≤ 25 year	4636.0	23.87 %
26~35 year	11329.0	58.33 %
36~45 year	2535.0	13.05 %
≥ 46 year	922.0	4.75 %
**Basic education level**		
Secondary diploma	4187	21.56 %
Associate degree	10,704	55.11 %
Baccalaureate degree	4531	23.33 %
**Highest education level**		
Secondary diploma	495	2.55 %
Associate degree	8234	42.39 %
Baccalaureate degree	10,664	54.91 %
Master’s degree/PHD	29	0.15 %
**Hospital level**		
Primary or community	316	1.63 %
Secondary	10,487	54.00 %
Tertiary	8619	44.38 %
**Working years**		
≤ 5 year	8788	45.25 %
6~15 year	8243	42.44 %
16~25 year	1523	7.84 %
≥ 26 year	868	4.47 %
**Nightshift status**		
Yes	10,846	55.84 %
No	8576	44.16 %
**Working department**		
Outpatient	2277	11.72 %
Emergency	1165	6.00 %
Impatient general ward	13,599	70.02 %
ICU	2381	12.26 %
**Marital status**		
Single	5577	28.71 %
Married	13,211	68.02 %
Widowed	47	0.24 %
Divorced	587	3.02 %
**Number of children**		
0	7138	36.75 %
1	7701	39.65 %
2	4507	23.21 %
3	76	0.39 %
**Living with parents**		
Yes	9852	50.73 %
No	9570	49.27 %
**Per capita income**		
≤550	690	3.55 %
551~1200	2185	11.25 %
1201~2000	3005	15.47 %
2001~3000	5025	25.87 %
3001~6000	6142	31.62 %
>6000	2375	12.23 %

### Distribution of mean scores of subscales and overall HPLP

In the current study, the mean score of self-actualization, health responsibility/physical activity, nutrition, job safety, interpersonal support, and overall HPLP were 27.61(5.42) out of a score of 36, 22.71(7.77) out of a score of 44, 10.43(2.97) out of a score of 16, 22.05(3.97) out of a score of 28, 20.19(4.67) out of a score of 28, and 102.99(19.93) out of a score of 144, respectively.

In the process of univariate analysis, all demographic data including gender, age, basic education level, highest education level, hospital level, working years, nightshift status, working department, marital status, number of children, living with parents, monthly income per person were analyzed. The outcome of the univariate analysis was presented in Table 2. The mean score of all subscales and the overall HPLP differed in participants with different ages, hospital level, working years, nightshift status, working department, marital status, and monthly income per person, and such differences were statistically significant (*P* < 0.05).

Table 2 also presented horizontal comparison in the mean score of 5 subscales and overall HPLP in different demographic characteristics. Regarding nurses’ age, we found that participants who aged more than 45 got a higher mean score of all 5 subscales and the overall HPLP than other age groups in the study. Such a difference was significant through pairwise comparison (*P* < 0.001). In terms of the highest education level shown in Table 2, the mean score of all subscales and overall HPLP was higher in participants with a postgraduate degree or above than that in nurses with other education backgrounds. Moreover, such differences in subscales and overall HPLP except in job safety were significant (*P* < 0.001). Additionally, higher mean scores were presented in participants working in Tertiary hospitals and the difference was statistically significant when performing pairwise comparison (*P* < 0.05). Moreover, the result showed a significant difference in the mean score of all subscales and the overall HPLP in subjects with different working years (*P* < 0.001). As seen in Table 2, nurses who worked for more than 26 years reported higher mean scores than other groups (*P* < 0.05). Compared with nurses who were not on nightshift rotation, nurses on nightshift rotation got lower mean scores in all subscales and the overall HPLP, and the statistical test indicated that such differences were significant (*P* < 0.001). Surprisingly, it was reported that widowed nurses got the highest mean score for each subscale and the overall HPLP in the current study. According to Table 2, there was a significant difference between the mean score of each dimension of the scale and the working departments (*P* < 0.001). Not surprisingly, nurses working in the ICU got the lowest mean score while nurses in the Outpatient got the highest mean score. As for monthly income per person, it was found that the higher income, the higher mean score in the study. Obviously, nurses with a monthly income per person>6000 Yuan got the highest mean scores for each dimension and the overall HPLP.

**Table 2:** Distribution of Subscales and overall HPLP

	self-actualization	health responsibility / physical activity	Nutrition	job safety	interpersonal support	Overall HPLP
**Gender**						
Male	28.00(5.57)	25.09(8.95)	10.31(2.98)	22.41(4.05)	20.65(4.76)	106.45(21.50)
Female	27.59(5.41)	22.60(7.69)	10.43(2.97)	22.03(3.97)	20.17(4.66)	102.83(19.84)
t	2.117	7.941	-1.202	2.646	2.94	4.796
*P*	0.034	<0.001	0.23	0.008	0.003	<0.001
**Age**						
≤ 25 year	27.11(5.41)	23.16(7.92)	9.92(2.95)	22.23(3.98)	20.64(4.69)	103.05(20.16)
26~35 year	27.51(5.44)	22.19(7.77)	10.23(2.95)	21.85(4.00)	19.86(4.69)	101.64(19.95)
36~45 year	28.38(5.20)	23.15(7.42)	11.52(2.74)	22.35(3.85)	20.46(4.51)	105.86(18.94)
≥ 46 year	29.19(5.18)	25.61(6.99)	12.40(2.50)	22.81(3.84)	21.26(4.31)	111.27(18.34)
F	61.446	77.314	387.735	28.877	3558.998	3561.706
*P*	<0.001	<0.001	<0.001	<0.001	<0.001	<0.001
**Basic education level**						
Secondary diploma	28.00(5.28)	22.80(7.35)	10.81(2.96)	22.07(3.87)	20.16(4.54)	103.84(19.17)
Associate degree	27.35(5.39)	22.60(7.85)	10.19(2.95)	21.96(4.00)	20.12(4.70)	102.22(20.06)
Baccalaureate degree	27.87(5.56)	22.90(7.95)	10.62(2.99)	22.23(4.00)	20.39(4.71)	104.01(20.24)
F	28.817	2.756	77.963	7.28	5.124	17.614
*P*	<0.001	0.064	<0.001	0.001	0.006	<0.001
**Highest education level**						
Secondary diploma	27.38(5.43)	23.10(7.10)	10.71(2.92)	21.87(4.09)	20.34(4.58)	103.40(19.32)
Associate degree	27.44(5.41)	23.07(7.93)	10.26(3.01)	22.08(4.01)	20.38(4.69)	103.23(20.29)
Baccalaureate degree	27.74(5.42)	22.41(7.65)	10.54(2.94)	22.03(3.94)	20.04(4.65)	102.76(19.67)
Master’s degree/PHD	29.62(5.49)	25.48(8.82)	12.52(3.00)	23.41(4.39)	21.79(4.74)	112.83(21.44)
F	6.355	12.31	19.947	1.715	9.712	2.961
*P*	<0.001	<0.001	<0.001	0.162	<0.001	0.035
**Hospital level**						
Primary or community	26.64(5.68)	22.25(7.77)	10.25(3.05)	21.63(4.11)	19.84(4.87)	100.60(20.67)
Secondary	27.19(5.37)	22.22(7.51)	10.21(2.90)	21.73(3.97)	19.83(4.61)	101.18(19.42)
Tertiary	28.15(5.41)	23.32(8.03)	10.69(3.03)	22.46(3.94)	20.65(4.69)	105.28(20.28)
F	80.004	47.695	63.286	83.569	74.579	102.561
*P*	<0.001	<0.001	<0.001	<0.001	<0.001	<0.001
**Working years**						
≤ 5 year	27.24(5.42)	22.90(7.90)	9.93(2.94)	22.03(4.05)	20.31(4.71)	102.41(20.18)
6~15 year	27.64(5.42)	22.05(7.67)	10.50(2.93)	21.87(3.94)	19.87(4.66)	101.95(19.75)
16~25 year	28.62(5.24)	23.56(7.43)	11.74(2.71)	22.60(3.77)	20.71(4.52)	107.23(18.80)
≥ 26 year	29.22(5.13)	25.57(6.98)	12.44(2.44)	22.82(3.79)	21.19(4.29)	111.25(18.18)
F	60.651	77.093	399.449	27.437	37.654	95.36
*P*	<0.001	<0.001	<0.001	<0.001	<0.001	<0.001
**Nightshift status**						
Yes	27.15(5.45)	22.41(7.87)	9.78(2.91)	21.86(4.01)	19.78(4.70)	100.97(20.11)
No	28.19(5.31)	23.10(7.62)	11.24(2.85)	22.30(3.92)	20.71(4.58)	105.54(19.40)
t	-13.386	-6.196	-35.138	-7.719	-13.873	-16.037
*P*	<0.001	<0.001	<0.001	<0.001	<0.001	<0.001
**Working department**						
Outpatient	27.98(5.33)	23.42(7.39)	11.39(2.90)	22.40(3.89)	21.02(4.54)	106.20(19.22)
Emergency	27.33(5.37)	22.86(7.80)	10.15(2.91)	21.99(3.90)	19.90(4.50)	102.23(19.60)
Impatient general ward	27.61(5.45)	22.65(7.79)	10.38(2.96)	22.01(4.01)	20.18(4.70)	102.82(20.06)
ICU	27.38(5.30)	22.33(7.90)	9.87(2.91)	22.00(3.85)	19.64(4.57)	101.22(19.71)
F	6.015	69.188	117.691	6.83	38.431	26.975
*P*	<0.001	<0.001	<0.001	<0.001	<0.001	<0.001
**Marital status**						
Single	27.13(5.44)	23.15(7.88)	9.88(2.92)	22.27(3.94)	20.57(4.65)	102.99(20.08)
Married	27.78(5.38)	22.50(7.71)	10.65(2.96)	21.94(3.99)	20.01(4.67)	102.88(19.85)
Widowed	28.89(5.13)	26.47(7.92)	11.43(2.83)	23.21(3.11)	21.91(3.80)	111.89(18.16)
Divorced	28.09(5.59)	23.06(7.69)	10.60(3.10)	22.46 (3.96)	20.51(4.67)	104.74(20.28)
F	21.565	13.167	92.192	12.479	22.057	4.772
*P*	<0.001	<0.001	<0.001	<0.001	<0.001	<0.001
**Number of children**						
0	27.20(5.44)	23.07(7.90)	9.93(2.93)	22.19(3.97)	20.55(4.65)	102.94(20.11)
1	27.81(5.43)	22.65(7.68)	10.69(2.98)	22.06(3.95)	20.09(4.64)	103.29(19.82)
2	27.90(5.32)	22.26(7.66)	10.76(2.92)	21.83(4.02)	19.80(4.71)	102.54(19.78)
3	28.79(5.19)	22.13(8.68)	10.83(3.05)	21.55(4.11)	19.82(5.01)	103.11(22.11)
F	22.913	10.438	106.93	8.016	26.651	1.38
*P*	<0.001	<0.001	<0.001	<0.001	<0.001	0.247
**Living with parents**						
Yes	27.62(5.43)	22.58(7.81)	10.46(2.97)	22.02(4.00)	20.18(4.69)	102.86(19.97)
No	27.59(5.41)	22.84(7.71)	10.39(2.97)	22.08(3.95)	20.21(4.64)	103.12(19.89)
t	0.357	-2.32	1.463	-0.939	-0.495	-0.892
*P*	0.721	0.02	0.143	0.348	0.621	0.372
**Monthly income per person**						
≤550	26.93(5.98)	22.63(8.34)	9.78(3.12)	21.29(4.33)	19.60(4.91)	100.23(21.76)
551~1200	27.00(5.45)	22.65(7.80)	9.90(2.93)	21.57(4.03)	19.69(4.72)	100.81(20.16)
1201~2000	27.37(5.28)	22.43(7.60)	10.09(2.85)	21.84(3.92)	19.83(4.64)	101.57(19.57)
2001~3000	27.40(5.38)	22.60(7.83)	10.26(2.98)	21.96(3.97)	20.04(4.66)	102.26(19.96)
3001~6000	27.78(5.38)	22.61(7.62)	10.62(2.94)	22.16(3.95)	20.34(4.60)	103.50(19.66)
>6000	28.61(5.39)	23.64(7.94)	11.35(2.94)	22.91(3.98)	21.24(4.60)	107.82(19.43)
F	29.921	7.782	83.489	37.849	37.731	42.01
*P*	<0.001	<0.001	<0.001	<0.001	<0.001	<0.001

### Results of category regression

In the process, all significant factors in the univariate analysis were included in the category regression in order to determine the correlation of the demographic data and the HPLP. The results were presented in Table 3. It was observed that gender, highest education level, hospital level, working years, nightshift status, number of children, and monthly income per person were included in the predicting equation of average score of self-actualization, and the equation was significant (Adjust R^2^ = 0.023, *P* < 0.001). And in those above factors, hospital level, nightshift status, monthly income per person, working years were the four most important factors which might impact the mean score of self-actualization with the importance rate, namely, 27.5 %, 25.6 %, 18 %, and 13.4 %. As for responsibility/physical activity, the predicting equation was also significant (Adjust R^2^ = 0.025, *P* < 0.001), in which all factors significant in the univariate analysis except living with parents were included. And age was the most important factor with an importance rate of 31.2 % that might impact the mean score of health responsibility/physical activity. In regard to Nutrition, all included factors significant in the univariate analysis were significant in the predicting equation for the mean score of nutrition (Adjust R^2^ = 0.093, *P* < 0.001), and nightshift status was found to be the most important factor with the important weight of 42.1 %. Regarding the mean score of job safety, factors including gender, hospital level, working years, nightshift status, working department, marital status, number of children and monthly income per person were included into the predicting equation, and such an equation was tested statistically significant (Adjust R^2^ = 0.024, *P* < 0.001). In such an equation, obviously, monthly income per person and hospital level were found as the two most important predicting factors for mean score of job safety. When exploring the predicting model for the mean score of interpersonal support, all included factors but working department were significant (Adjust R^2^ = 0.042, *P* < 0.001). As shown in Table 3, it is evident that nightshift status (Importance weight: 24 %) and monthly income per person (Importance weight: 20.5 %) were the two most important predictive factors for interpersonal support. With regard to the total score of HPLP, gender, age, basic education level, highest education level, hospital level, working years, nightshift status, working department, marital status, and monthly income per person were predictive variables in the significant equation (Adjust R^2^ = 0.039, *P* < 0.001), and the results indicated that nightshift status (25.6 %), hospital level (22.2 %), working years (21.8 %) and monthly income per person (17.9 %) were the four most important predictive factors.

**Table 3:** Outcome of Category Regression (Importance)

β						
	**self-actualization**	**health responsibility and physical activity**	**Nutrition**	**job safety**	**interpersonal support**	**Overall HPLP**
**Gender**	0.025(1.6 %) *	-0.066(16.7 %) *	**------------------**	0.015(1.1 %) *	0.021(1.0 %) *	0.043(3.9 %) *
**Age**	0.018(6.7 %)	0.092(31.2 %) *	0.076(17.5 %) *	-0.036(3.6 %)	-0.047(5.9 %) *	-0.031(0.1 %) *
**Basic education level**	0.013(1.4 %)	**------------------**	0.049(-0.007 %) *	0.015(0.6 %)	0.040(1.8 %) *	0.041(1.6 %) *
**Highest education level**	0.013(0.7) *	-0.037(6.0 %) *	-0.024(-1.1 %) *	**------------------**	-0.059(4.9 %) *	-0.046(1.3 %) *
**Hospital level**	0.073(27.5 %) *	0.068(18.8 %) *	0.052(4.5 %) *	0.079(28.4 %) *	0.082(16.5 %) *	0.089(22.2 %) *
**Working years**	0.037(13.4) *	-0.043(3.6 %) *	0.079(18.1 %) *	0.042(9.8 %) *	0.031(4.0 %) *	0.080(21.8 %) *
**Nightshift status**	0.064(25.6 %) *	0.042(7.3 %) *	0.162(42.1 %) *	0.051(11 %) *	0.105(24.0 %) *	0.092(25.6 %) *
**Working department**	0.015(0.5 %) *	-0.023(2.8 %) *	-0.056(7.5 %) *	0.014(1.7 %)*	-0.048(8.1 %)	-0.033(4.6 %) *
**Marital status**	0.009(1.9 %)	-0.024(4.1 %) *	0.017(0.7 %) *	0.053(8.7 %) *	0.027(3.4 %) *	0.035(1.0 %) *
**Number of children**	0.020(2.7 %) *	-0.035(4.8 %) *	0.019(1.3 %) *	-0.016(2.2 %) *	-0.069(10.0 %) *	**------------------**
**Living with parents**	**------------------**	0.013(-0.008 %)	**------------------**	**------------------**	**------------------**	**------------------**
**Monthly income per person**	0.05(18 %) *	0.033(5.8 %) *	0.067(10.1 %) *	0.087(32.9 %) *	0.091(20.5 %) *	0.072(17.9 %) *
**R**	0.155	0.16	0.306	0.16	0.208	0.203
R^2^	0.024	0.026	0.094	0.026	0.043	0.041
Adjust R^2^	0.023	0.025	0.093	0.024	0.042	0.04
*P*	<0.001	<0.001	<0.001	<0.001	<0.001	<0.001

备注: *:*P*<0.05

## Discussions

In recent years, HPLB continue to attract researchers’ attention. Nevertheless, there is limited evidence on HP in clinical nurses even though they are in a unique position in the healthcare system. Thus, our current study was designed to examine the HPLB and to explore some relative predictors for HPLB in clinical nurses in China.

In the current study, the mean score of self-actualization, health responsibility/physical activity, nutrition, job safety, interpersonal support, and overall HPLP were, respectively, 27.61(5.42), 22.71(7.77), 10.43(2.97), 22.05(3.97), 20.19(4.67) and 102.99(19.93), which indicate a moderate level of health promoting behaviors in Chinese clinical nurse. Several previous studies [7, 8, 13–15, 21] have examined HPLB in nursing students, and their results were not consistent with our study. On one hand, the subjects we assessed were different. On the other hand, measurements used in those studies differed from those used in the current study. For example, clinical nurses are the ones who offer healthcare for their clients directly while most nursing students do not. The measurement used in our study includes a subscale of job safety. For those reasons, the findings in the current study are different from previous studies.

There is an ongoing debate on the predictors for the HPLB. In the current study, we found nightshift status was one of the main predictors of overall HPLB and subscales scores. In China, most nurses on nightshift rotation works in 3 shifts: 8am-4pm, 4pm-12mn, 12mn-8am. Usually, nurses have at least 2 days of shift working in a week. Such shifts could offer continuous care services for patients. Unfortunately, internal circadian rhythms are disrupted by those irregular working hours, which leads to sleep disorders, irregular eating habits, irregular exercise, or less motivation/empowerment to make healthier lifestyle plans [22]. For example, nurses on the night shift working are more likely to have irregular meal frequency or to skip meals [23]. Even worse, physical and mental tiredness due to nightshift rotation would directly or indirectly increase the consumption of cigarettes, alcohol, junk food [22], fast food, higher calories food, and a higher amount of snacks [23]. On one hand, they have less time to prepare a healthy diet since most of their time is spent in recovering from such irregular shift rotation. On the other hand, it is much more easily accessible and cheaper to have junk food or snacks than to have a healthy and balanced diet [23], especially at night during nurses on their night shift working. As a result, they may be exposed to inadequate fruit and vegetable intakes. In terms of physical activities and interpersonal support, irregular rotating shift work made it difficult to participate in leisure-time physical activities because they had to spend more time recovering from such an irregular rotation working schedules. As a result, they had less time or energy to hang out with family/friends or to participate to exercise schedules or other stress-reduction behaviors [24, 25]. Based on the result of the current study, we suppose that leaders should pay more attention to taking interventions to empower or motivate nurses to maintain healthy diet habits and exercise, especially focus more on shift nurses’ health promoting lifestyle behaviors. Nurse managers should also take it into consideration to arrange nightshift in a more optimal way.

Notably, the current study points out the importance of hospital level in predicting the score of subscales and the overall HPLP. Contrary to the results of Wang [26], we surprisingly found that nurses working in the tertiary hospital got the highest mean score in all subscales and the overall HJPLP. In China, hospitals are classified into 3 levels including primary level, secondary level and tertiary level. Evidence showed that nurses in the tertiary hospitals faced with a much higher level of workload [27, 28] in China because patients preferred seeking healthcare at higher level hospitals [29]. As a result, nurses in the higher level of hospital deliver wider and more comprehensive health care services to clients [30]. However, this phenomenon makes nurses face more health-related issues, which might lead them more likely to reflect on health and health promotion. Therefore, they might pay more attention to health responsibility, physical activity, and nutrition. Additionally, evidence shows that, in China, nurses in a higher level of hospitals get much more payment than those in the primary care settings [31], which allows them to behave healthier such as buying quality healthy food, exercising in the gym [26]. In fact, one of the main findings in the study was the significant relationship between income and HPLB. Specifically, higher income, higher level of HPLB, which significantly supports that improving nurses’ welfare/income might improve their HPLB. Moreover, higher level hospitals have more opportunities for continuing education [32] and support channels. In other words, nurses in a higher level of hospitals gain more opportunities to receive health-related education and to seek personal support.

Surprisingly, we found that younger nurses got the lower score while nurses over 45 years got the best performance in the survey of subscales and overall HPLB in the current study, which was different from the opinion of Conner and Norman [33]. There are limited studies on the relationship between age and HPLB. The Reason why older nurses perform better in the survey, we suppose, are as follows. Firstly, we surprisingly found that age is strongly associated with the score of nutrition in the study. In other words, age was negatively correlated with the score of nutrition. Specifically, the younger nurses were, the lower scores of nutrition they got. We suppose that, on one hand, younger nurses face frequent night shift working due to nurse staffing inadequacy in China, which leads to unhealthy eating habits. On the other hand, the culture of body image deeply impacts younger nurses. Traditionally, it has been one of the most important parts of feminine identities to keep women’s physical beauty [34]. However, the cultural standard of ideal beauty has changed from traditional mild plum bodies to thin images under the influence of the economy and cultural transformation across cultures. Evidence showed that it had been a phenomenon of pathological weight concerns [34]. Comparing with older nurses, younger nurses are more over-conscious about their body image, and there is increasing body dissatisfaction among this young nurse group. The value of “the thinner, the better” results in eating disorders such as anorexia nervosa [35] or unhealthy eating habits behaviors in order to be thin. For instance, they would be on a diet, or have diets without five groups of nutrition. Regarding self-actualization, it contains self-value, self-achievement, and self-identity. In other words, professional identity is one of the most important dimensions of self-actualization. Professional identity does not always stain the same during nurses’ careers. In fact, it has changed over time. According to a report about nurses’ development status in China published in 2017 [36], 49.7 % of nurses were not clear about their career choice. Particularly, the feeling of burnout was the strongest during the very beginning of 6 to 10 years being a clinical registered nurse which may lead to a high level of turnover intention. However, compared with younger nurses, older nurses accumulate more clinical experience, become more skilled, and gain more confidence during their career, which might increase their professional identity [37]. In terms of health responsibilities/physical activities, theoretically, younger nurses should be more energetic to participate in physical activities than older ones. In fact, younger nurses usually face more challenges and stress comparing with older ones. On one hand, they are less experienced, and they are required to be trained in order to enhance their competencies. Especially, every novice has to receive standardized in-service training in China [38]. That is to say, younger nurses have less leisure time to involve in physical activities. On the other hand, the huge shortage of nurses results in heavy burden and overwork in clinical registered nurses, which is more of concern in younger nurses. That heavy workload would impact on the HPLB in nurses was strongly supported by another finding, nurses working in the emergency and ICU where nurses face with more challenges and workload got lower scores of subscales and overall HPLB in the current study. Overall, lower professional identity, and overwork, and frequent night shift rotation due to lack of nursing staff prevent younger nurses from promoting healthy lifestyle behaviors.

## Limitations of the study

The main limitation of the study is the design of a cross-sectional study. Due to the nature of the design, we could only access the data at one point in time. Besides, we could not gain more and further data, especially about nurses’ feelings or experiences on HPLB because of lacking qualitative evidence in this study. The mixed methodology could be used in future studies to take deeper insight of nurses’ HPLB. Moreover, bias might occur when participants complete such a self-reporting questionnaire.

## Conclusions

The study aimed to examine the HPLB of Chinese registered nurses and explore relative predictors. The results indicate that nurses who participated in the study presented a moderate level of all subscales and overall HPLP. Hospital levels, nightshift status, age, and monthly income per person were predictors for all subscales and overall HPLP. This study suggests that there is an urgent need that nursing leaders and managers should focus more on nurses’ HPLB, and take measures to solve the dilemma of shortage of nurse staffing, to arrange night shift work schedules in a more reasonable way, to improve nurses’ income, and to address correct value about body image.

## Data Availability

All the data supporting the study findings are within the manuscript. Additional detailed information and raw data are available from the corresponding author on reasonable request.

## Abbreviations

HP: Health Promotion
WHO: World Health Organization
HPLB: Health Promoting Lifestyle Behaviors
HPLP: Health Promoting Lifestyle Profile
M (SD): Mean (standard deviation)
